# A Monte Carlo Study of the Photon Spectrum due to the Different Materials Used in the Construction of Flattening Filters of LINAC

**DOI:** 10.1155/2017/3621631

**Published:** 2017-07-10

**Authors:** J. S. Estepa Jiménez, M. Díaz Lagos, S. A. Martinez-Ovalle

**Affiliations:** ^1^Universidad Nacional de Costa Rica (UNA), Avenida 1, Calle 9, Apartado Postal 86-3000, Heredia, Costa Rica; ^2^Universidad Pedagógica y Tecnológica de Colombia, Boyacá, Colombia; ^3^Clínica Cancerológica de Boyacá, Tunja, Colombia

## Abstract

Different types the spectrum of photons were studied; they were emitted from the flattening filter of a LINAC Varian 2100 C/D that operates at 15 MV. The simplified geometry of the LINAC head was calculated using the MCNPX code based on the studies of the materials of the flattening filter, namely, SST, W, Pb, Fe, Ta, Al, and Cu. These materials were replaced in the flattening filter to calculate the photon spectra at the output of this device to obtain the spectrum that makes an impact with the patient. The different spectra obtained were analyzed and compared to the emission from the original spectra configuration of the LINAC, which uses material W. In the study, different combinations of materials were considered in order to establish differences between the use of different materials and the original material, with the objective of establishing advantages and disadvantages from a clinical standpoint.

## 1. Introduction

The need to know with precision the photon spectrum that is emitted from the linear accelerators in clinical use (LINAC) for the different treatments of cancer is important for both the manufacturers and experts in clinical dosimetry. Companies such as Varian [1] and Elekta [2], among others, have been developing and improving their different models, offering new possibilities as technology continues to advance. The peculiarity of the construction of these machines lies in the selection of materials that comply with the quality standards according to their function as part of the machine. This study analyzes the answer regarding the photon beam produced by the flattening filter when different materials are combined in its construction.

The Monte Carlo method is considered an adequate tool for the study and modeling of radiation transport through different media. This method, although complex, takes into account all of the possible physical phenomena that occur during the interaction between the radiation and the matter [3].

In radiation therapy treatments with high energy LINAC (>10 MV), the inevitable production of neutrons occurs and contributes an additional dose to the patient due to the materials used [4]. Therefore, these neutrons are produced as a result of the interaction with the photon beam and the material used for the construction of the collimators (primary and secondary), the jaws, the target where the electron beam makes impact, and the flattening filter that has direct interaction with the beam.

The flattening filter of a LINAC is an important element in these devices, as it permits the determination of the most appropriate photon spectrum in cancer treatments. Research conducted regarding this component shows the relevance of determining its characteristics and also raises new questions. The work of Podgorsak et al. [5] demonstrates deep-dose measurements with an acrylic phantom used with targets and flattening filters of different materials. The results indicate that when using flattening filters with high-*Z* material, the beam has a flat form, though when using filters with low-*Z* material, the flat form is less prominent. Specifically, the spectrum of X photons is shown to be more penetrating when the target is combined with a flattening filter with Al, while the spectrum of X photons combined with a Pb target and flattening filter makes the beam less penetrating.

Also, Van Laere and Mondelaers [6] simulated a linear accelerator with operating energies of 5 and 10 MeV. In this experiment, they designed Pb conic flattening filters of different sizes over a 3 mm thick Al support. The results showed not only that the filters flattened the beam as a function of the design of the flattening filter but also that they were also able to differentiate the fluence spectrum depending on the energy at which the LINAC operated. On the other hand, Waggener et al. [7] measured the attenuation of the primary spectrum of X-rays for an energy of 18 MV using filters made of Al and Cu and made comparisons between the measured and the calculated curves of the transmission. Through the work of Iwasaki et al. [8], it is reported that modifications were made in the method of estimation of high energy X-rays proposed by Waggener et al. [7], with aims to minimize the differences between the measured spectrum and the calculated spectrum, with the variant that this final method was performed outside of the axis of the beam. In this experiment, low density materials were used for different filters and attenuators, and a Mitsubishi EXL-20DP/20TP LINAC was used to produce X-rays of 4.0, 10.0, and 15.0 MV. The materials used in the flattening filter for the X-rays of 4 MV were Cu (59–62%), Pb (<0.1%), Fe (<0.1%), and Zn (38–41%); the filter for the 10 MV X-rays was composed of W (65%) and Cu (35%); and the filter for the 15 MV X-rays was made up of W (90%), Mo (4%), Fe (2%), and Ni (4%).

In the studies of Martínez-Ovalle et al. [4], the dose calculations were reported to be equivalent due to the production of neutrons in targets and flattening filters of the linear accelerators. The results indicate that the high-*Z* materials produce a higher quantity of neutrons and consequently a higher additional dose to the patient due to the neutrons. Furthermore, Kragl et al. [9] studied the impact to the dose due to the flattening filter, while Hrbacek et al. [10] propose the design for a new linear accelerator with a beam-type flattening filter, modeled by way of a mathematical algorithm.

Also experiments of Vassiliev et al. were found [11], in which they study the basic dosimetry properties of a photon beam of 6 MV and of 18 MV for a Varian Clinac 21EX operating without a flattening filter and compare the properties with measurements of photon beams. When the flattening filter is removed, the contamination stemming from the electrons and neutrons (*E* > 10 MV) changes, because the photons give up their energy due to interaction with the atoms of the material of the flattening filter [12]. There are benefits to removing the flattening filter, such as an increase in the dose rate, reduction of the dispersion, reduction of radiation leakage, and reduction of the out-of-field dosage [13].

The flatness of the beam that impacts the patient is fundamental, as demonstrated by Vassiliev et al. [11], because as the flattening filter is removed, the quantity of photons dispersed in the head is reduced, and, consequently, a reduction in the dose in the healthy tissues and organs, which affects the organ to be treated due to the inhomogeneous distribution of the dose. This problem can only be solved with the inclusion of the filter. In this context, Stathakis et al. [14] studied the dose received by patients who are subjected to intensity modulated therapy with and without the filter.

Due to the aforementioned, the study of the flattening of the beam in these pieces of equipment is relevant due to the effect that this may have on the dose that the patient receives. The main objective of this calculation is to study the photon spectrum that is emitted from the head of the LINAC and establish the differences in the different emission spectra as the material of the flattening filter is changed.

Although there are studies in which the flattening filter is removed, especially in small fields used in SRS (Stereotactic Radio Surgery), it is clear that for larger fields, such as those used in IMRT (Image-Guided Radiation Therapy), IGAT (Image-Guided Adaptive Radiation Therapy), SBRT (Stereotactic Body Radiation Therapy), or (VIMAT) Volumetric Intensity Modulated Arc Therapy, the flattening filters are needed [15].

Other studies show that there are significant differences in dose calculations using flattening filter and calculations without flattening filter [16]. These differences are consistent with the results found in this work, where there is a reduction in photon flux up to 98% with the inclusion of the flattening filter and low-energy photon beam filtering is effective. It is clear that there are crucial differences with the inclusion or not of the flattening filter and although it is considered that there are improvements without flattening filter, in the majority of applications its inclusion is necessary [17].

## 2. Materials and Methods

**Figure 1:**
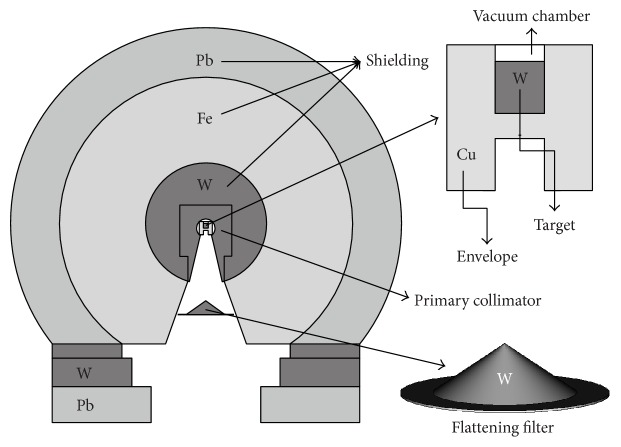
Geometry of the head of the LINAC with the original materials used by this model in each component.

The geometry was constructed of a LINAC Varian Clinac 2100 C/D operating at 15 MV in compliance with manufacturer's specifications, following the instructions of Mao et al. [19]. Shown in Figure 1 is the geometry of head of the LINAC that was simulated, in which the main components of the head stand out.

The target in this LINAC was simulated with a cylinder with a radius of 0.301 cm and a height of 0.635 cm made of the material W in mind. In the top part of the target, an empty space with a height of 0.2 cm was considered, in which the electrons impact the target. In this study, the beam of electrons that impacts the target is simulated by a 15.04 MeV monoenergetic beam, which is determined once the tuning process is performed upon adjusting the experimental PDD with the calculated PDD. The number of histories simulated was 1 · 10^7^ in a parallelized cluster with an uncertainty of 0.5%.

The envelope is the coating that is found embedded in the target, which is often of Cu, whose function is to serve as a support to the target as well as a refrigerant. The primary collimator is simulated with a concentric sphere with a radius of 8.3 cm made of the material W. This component has conical opening that projects a field of 50 cm in diameter to the height of the isocenter, and its function is to prevent the leakage of photons dispersed in the head and to limit the size of the field of the beam. The shielding of the LINAC was constructed using concentric spheres with radii of 20.1 cm and 26.7 cm. (See Figure 1). Each of the spheres contains high-*Z* materials that permit the reduction of the radiation dispersed from the target.

The flattening filter, the device of interest in this study, was designed considering a cylinder with a radius of 3.81 cm and a truncated cone with a height of 1.89 cm. The materials used for the filter were taken from that reported in the literature, with the clarification that the manufacturing companies omit details and characteristics, which makes this type of studies difficult. The relation of the materials found is shown in Table 1.

**Figure 2:**
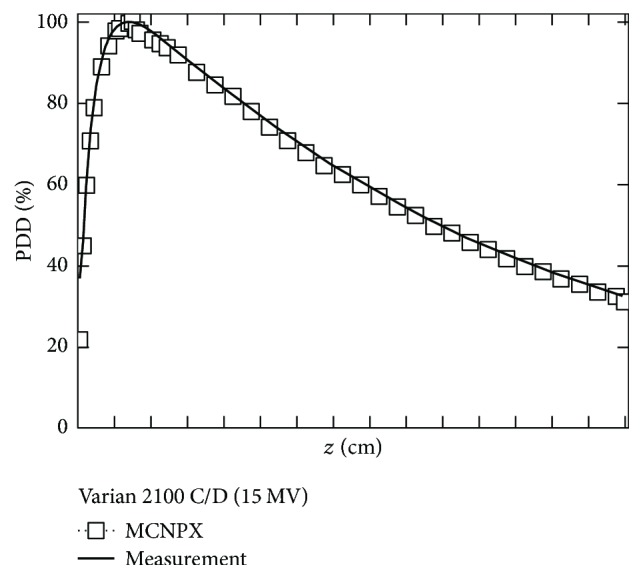
Comparison of PDD calculated with MCNPX with experimental measurements for the accelerator Varian Clinac 2100 C/D 15 MV.

The first step of the simulation was to fix the characteristics of the electron beam impinging the target of LINAC. We assumed a point-like source emitting electrons in a single direction; the tuning curve is shown in Figure 2. The tuning was carried out by assuming that initial electrons followed a Gaussian energy distribution and fixing the mean energy and FWHM values of this Gaussian to reproduce, as usual, the value of TPR_20,10_, that is, the ratio of the doses at 20 and 10 cm in water and in the beam axis, with a distance between the source and the detector fixed to 100 cm and a field of 10 · 10 cm^2^ at the surface of the detector. Were simulated 5 · 10^10^ histories, using a multiprocessor computer to obtain stochastic uncertainties <3%.

**Figure 3:**
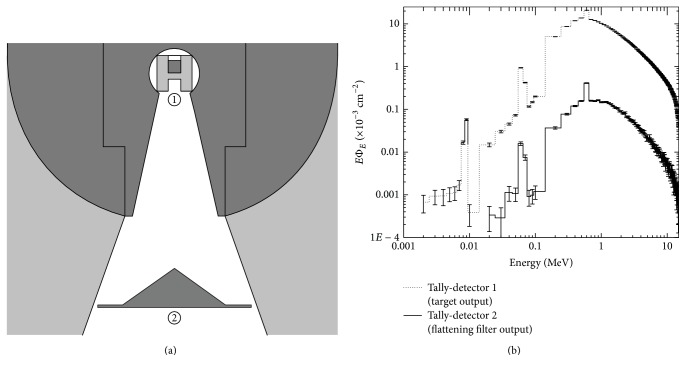
Calculation points located at the output of the target and the output of the flattening filter (panel (a)). Fluency spectrum of photons per electron emitted in detector tallies 1 and 2 (panel (b)).

**Figure 4:**
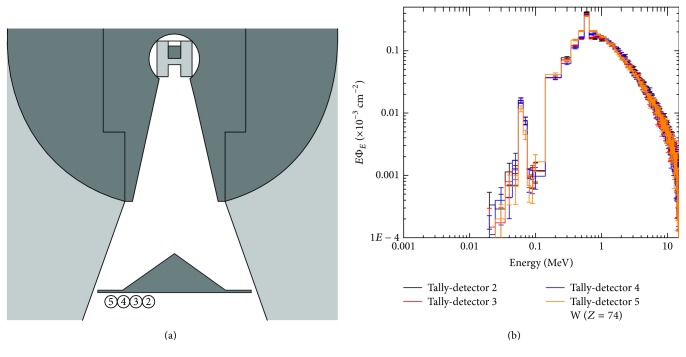
Detectors located to analyze the variation of the fluence spectrum at the output of the flattening filter (a). Photon spectrum at detector tallies 2, 3, 4, and 5 for W (b).

The next step consists in stablishing the location of the tally detectors at the points of interest of the geometry under study, in this case, our LINAC Varian 2100 C/D. The first point of interest is the exit of the target; there is located a detector tally (1) of 0.5 cm in diameter, as seen in Figure 3(a). The next point of interest is the exit of the flattening filter, where 4 detectors tally (2, 3, 4, and 5) of 0.5 cm in diameter were located from the central axis to the edge of the flattening filter, as seen in Figure 4(a).

## 3. Results and Discussion

To obtain the photon spectra that are emitted from the LINAC, detector tallies were placed at different points inside the head of the LINAC, specifically at the output of the target and at the output of the flattening filter. It is clarified that, for the respective calculations, the materials of the flattening filter shown in Table 1 were replaced, and the same procedure was followed for each of the materials.

Firstly, the tuning energy for the Varian Clinac 2100 C/D LINAC was established, for which measurements were taken in an H_2_O phantom with aims to obtain PDD. The same process was carried out using the Monte Carlo method until the PDD were adjusted. The maximum dose, due to the photons in the build-up region, is 4.85 · 10^−16^ Gy of photons per electron emitted from the source, and the placement corresponds to 3.0 cm of depth in a water phantom. Shown in Figure 2 is the tuning curve.

Next, the attributes of the photon beam emitted from the Varian Clinac 2100 C/D at 15 MV were determined, which corresponds to the original geometry according to the manufacturer. Figure 3(a) shows an enlarged image of the area where the target was placed (the area where the electron beam impacts) and the flattening filter (device that flattens the beam). The company Varian uses material W for the target because it satisfies two fundamental conditions: a high atomic number (*Z*), which maximizes the efficiency of the production of X-rays, and a high melting point, which minimizes damage caused by the electron beam [20].

In Figure 3(b), it is shown that the photon fluence spectrum is emitted from the target and the flattening filter. From the observation of the spectrum in detector tally 1, a characteristic spike is evident at 0.511 MeV, which may be associated with the photons resulting from annihilation and a spectrum of X photons with energies mainly between 0.1 and 15 MV. The main observation with respect to detector tally 2 is that the photon fluence is reduced by ≈98% in relation to the fluence emitted from the target (detector tally 1), and the spike of maximum fluence is maintained, with a tendency to increase, due to the production of photons as a result of annihilation. When the two spectra are compared, observing detector tally one, it is found that characteristic spikes occur, which coincide with the information reported by Israel et al. for low-energy X photons [21].

From detector tally 2, it is observed that, for energy values of less than 0.02 MeV, the photons are absorbed by the flattening filter, allowing only higher-energy X photons to emerge from the other side of the flattening filter. The photon spectrum emitted from the filter maintains the same form, and the characteristic spikes maintain the same range of energy that is emitted from the target. This characteristic is related to the materials of the target and the filter, which are the same in this case.

Changes that the beam presents at the different flattening filter positions were also verified, for which detector tallies were placed from the axis of beam to the edge of the flattening filter as shown in Figure 4(a). The comparative spectrum that was obtained from four detector tallies is shown in Figure 4(b).

From the analysis of Figure 4, it is inferred that the peak of the maximum fluence that corresponds to the photons resulting from annihilation with an energy of 0.511 MeV maintains the same distribution in the four spectra, and they are also found to be within the same ranges energy. Nevertheless, the photon fluence appears to decrease as we move further away from the beam axis, since the thickness decreases with the distance of the axis of the beam due to the geometry of the flattening filter. At times, the orange one corresponds to the highest values.

**Figure 5:**
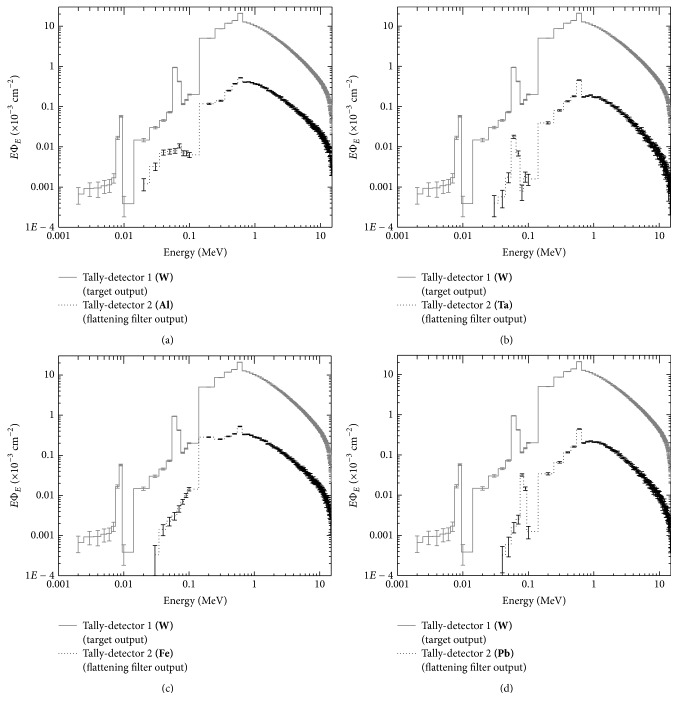
Spectrum of W at the output of the target compared to the output of the flattening filter for Al panel (a), Ta panel (b), Fe panel (c), and Pb panel (d).

**Figure 6:**
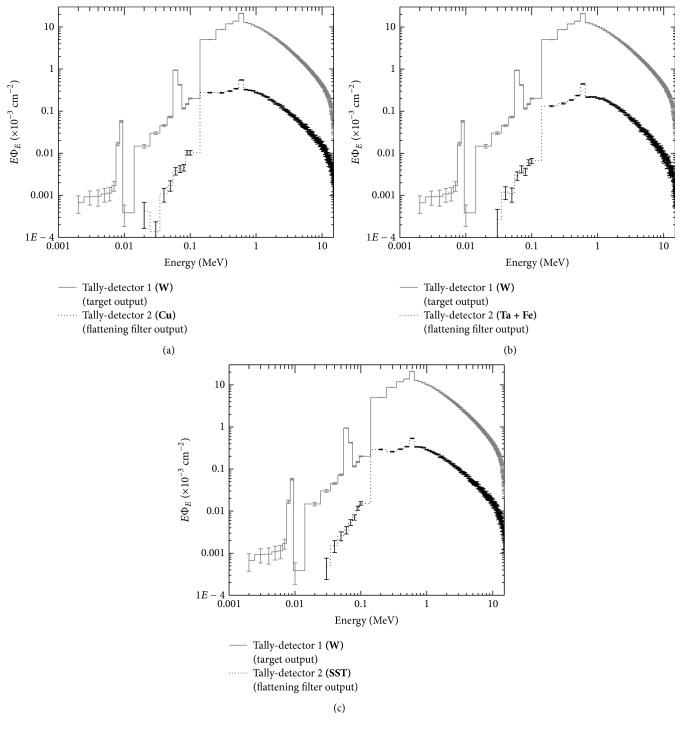
Spectrum of W at the output of the target compared to the output of the flattening filter for Cu panel (a), Ta + Fe panel (b), and SST panel (c).

Because the main objective of this study is to determine the characteristics of photon beams as a function of the inclusion of different materials of the flattening filter, the material of the flattening filter was modified with the materials established in Table 1. All of the geometry of the original Varian head was maintained. Separate calculations were made for each material, though the target material was conserved (W). In Figures 5 and 6, the obtained results are shown.

From the observation of the spectra shown in Figures 5 and 6, it is established that they have very similar characteristics, particularly that they all presented a characteristic spike of photons resulting from annihilation with an energy of 0.511 MeV, and this is because the photon spectrum that is omitted from the target is the same. For low-*Z* materials, increased particles flow across the fluence spectrum occurs because their atomic spacing is more separated, and the cross section of the interaction is much less. High-*Z* materials allow greater absorption of low-energy X photons, allowing photons of certain energies that will be useful treatment to pass through. It is also evident that if the material has approximately the same* Z*, the form of the spectrum is similar, which indicates that the photon fluence depends on the* Z* of the material.

**Figure 7:**
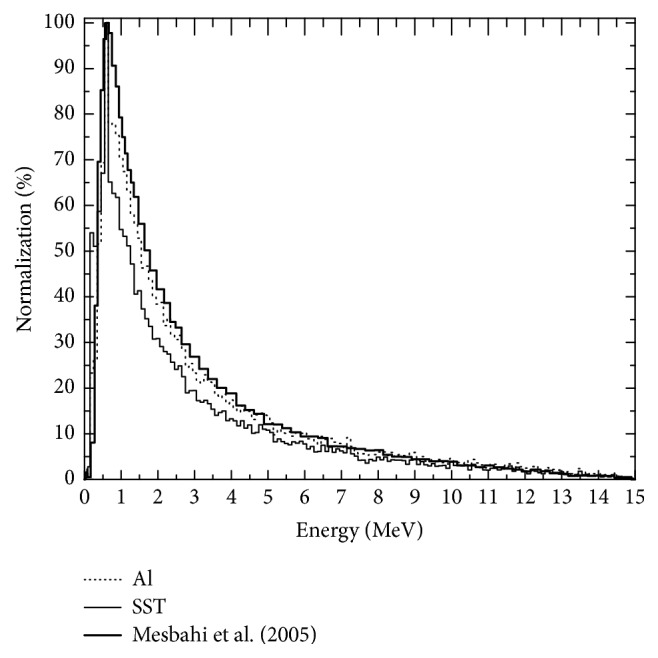
Comparison of normalized spectra obtained by Mesbahi et al. [18] and the spectra obtained in this study.

In Figure 7, the fluence spectra for Al and SST are compared to those reported by Mesbahi et al. [18]. They characterized the photon beam of a Varian Clinac 2300 C/D with codes MCNP4C y GEANT3 and simulated the accelerator head, bearing in mind the factory specifications, and made the comparison of the distribution of the dose over the water phantom for different sizes of radiation fields for photon fluence spectra with accelerators operating at 6 MV and 15 MV. The spectrum calculated at 90 cm from the target (SSD = 90 cm) is what was compared with the value over the radiation axis at detector tally 2 of materials Al and SST obtained in the study. Although the points are not the same, the comparison can be carried out considering that the loss of energy of the photons in 78 cm of air is minimal, comparing the distance between the flattening filter and the point calculated by Mesbahi et al. [18] (90 cm).

In Figure 7, some differences that are likely associated with the simulated model of the LINAC are shown. Although they are both Varian, they are different versions, and there is a lack of clarity in the materials used. In our case, the different parameters are well-defined. In the spectra for the Al and SST, a high coherence exists in spite of the fact that the material used drastically influences the spectrum, as previously mentioned.

## 4. Conclusions

We were able to determine that photons with energies of less than 0.2 MeV are attenuated due to photoelectric absorption in the flattening filter, as they must travel distance of at least as large as that of the difference in the thickness of the filter and the electron range that is emitted from the target.

The photon fluence that emerges from the flattening filters depends directly on* Z*; nevertheless, this aspect may not be relevant if the large size of the pulsed beam that these accelerators emit is considered.

With respect to the materials, it is concluded that low-*Z* materials present higher fluence, as the atomic spacing is not found to be as close together as those of high-*Z* materials. There are advantages and disadvantages that are related to the type of treatment performed on the accelerator; low-*Z* materials are used in linear accelerators for treatments with electrons, while high-*Z* materials are used for treatments with photons.

This research in combination with other results could validate the capacity of the simplified applied beam models as long as the dependence of simulated energy spectra and quality radiation is small compared to their energy range.

## Figures and Tables

**Table 1:** Materials used for the construction of flattening filters in linear accelerators.

Element	*Z*	*A*	%	*ρ* [g·cm^−3^]	References
Al	13	27	100	2.698	(i) Podgorsak et al. (1974)
					(ii) Waggener et al. (1999)
Fe	26	56	100	7.874	(i) Mao et al. (1997)
					(ii) Iwasaki et al. (2003)
Cu	29	64	100	8.96	(i) Waggener et al. (1999)
					(ii) Iwasaki et al. (2003)
Ta	73	181	100	16.654	(i) Martínez-Ovalle et al. (2011)
W	74	184	100	19.3	(i) Iwasaki et al. (2003)
					(ii) Martínez-Ovalle et al. (2011)
Pb	82	208	100	11.4	(i) Podgorsak et al. (1974)
					(ii) Van Laere and Mondelaers (1997)
					(iii) Iwasaki et al. (2003)

SST (Cr/Fe/Ni)			18/74/8	8.03	(i) Martínez-Ovalle et al. (2011)
					(ii) González et al. (2009)
Ta + Fe			100/100	16.654/7.874	(i) Martínez-Ovalle et al. (2011)
					(ii) Mao et al. (1997)
